# A Novel Ten-Gene Signature Predicting Prognosis in Hepatocellular Carcinoma

**DOI:** 10.3389/fcell.2020.00629

**Published:** 2020-07-14

**Authors:** Taicheng Zhou, Zhihua Cai, Ning Ma, Wenzhuan Xie, Chan Gao, Mengli Huang, Yuezong Bai, Yangpeng Ni, Yunqiang Tang

**Affiliations:** ^1^Department of Gastroenterological Surgery and Hernia Center, The Sixth Affiliated Hospital of Sun Yat-sen University, Guangdong Institute of Gastroenterology, Guangdong Provincial Key Laboratory of Colorectal and Pelvic Floor Diseases, Supported by National Key Clinical Discipline, Guangzhou, China; ^2^Department of Oncology, The Affiliated Cancer Hospital of Guangzhou Medical University, Guangzhou, China; ^3^The Medical Department, 3D Medicines Inc., Shanghai, China; ^4^Department of Oncology, Jieyang People’s Hospital, Sun Yat-sen University, Jieyang, China; ^5^Department of Hepatic-Biliary Surgery, The Affiliated Cancer Hospital of Guangzhou Medical University, Guangzhou, China

**Keywords:** hepatocellular carcinoma, expression, prognosis, signature, risk stratification

## Abstract

Hepatocellular carcinoma (HCC) has a dismal long-term outcome. We aimed to construct a multi-gene model for prognosis prediction to inform HCC management. The cancer-specific differentially expressed genes (DEGs) were identified using RNA-seq data of paired tumor and normal tissue. A prognostic signature was built by LASSO regression analysis. Gene set enrichment analysis (GSEA) was performed to further understand the underlying molecular mechanisms. A 10-gene signature was constructed to stratify the TCGA and ICGC cohorts into high- and low-risk groups where prognosis was significantly worse in the high-risk group across cohorts (*P* < 0.001 for all). The 10-gene signature outperformed all previously reported models for both C-index and the AUCs for 1-, 3-, 5-year survival prediction (C-index, 0.84 vs 0.67 to 0.73; AUCs for 1-, 3- and 5-year OS, 0.84 vs 0.68 to 0.79, 0.81 to 0.68 to 0.80, and 0.85 vs 0.67 to 0.78, respectively). Multivariate Cox regression analysis revealed risk group and tumor stage to be independent predictors of survival in HCC. A nomogram incorporating tumor stage and signature-based risk group showed better performance for 1- and 3-year survival than for 5-year survival. GSEA revealed enrichment of pathways related to cell cycle regulation among high-risk samples and metabolic processes in the low-risk group. Our 10-gene model is robust for prognosis prediction and may help inform clinical management of HCC.

## Introduction

Hepatocellular carcinoma (HCC) is the sixth most common cancer and the fourth leading cause of cancer mortality worldwide (Bray et al., 2018). While the vast majority of HCC patients are no longer eligible for curative therapy at the time of diagnosis, those who have undergone curative resection or transplantation still face a 70% risk of recurrence in 5 years (Bruix et al., 2014; Mazzaferro et al., 2014; European Association for the Study of the Liver. Electronic address: easloffice@easloffice.eu, and European Association for the Study of the Liver, 2018). The dismal prognosis of HCC can be attributed to a number of factors and there exists a demand for a model that can effectively identify patients at a high risk of recurrence/metastases so that clinical actions could be taken proactively. Conventional prognostic models for HCC mainly involve integration of clinicopathological factors such as tumor size, number of lesions, microvascular invasion, and cirrhosis, supplemented by serum levels of certain single markers such as α−fetoprotein (AFP) and des−gamma carboxy−prothrombin (DCP) (Marrero et al., 2009; Chan et al., 2018). However, their specificity and sensitivity do not support distinguishment of meaningful patterns of prognosis, especially with the substantial heterogeneity of HCC.

With the advent of massively parallel sequencing, molecular characterization has identified key driver pathways in HCC and several schemes for subtyping HCC have been proposed according to genomic, transcriptomic, microRNA (miRNA), and proteomic profiles (Boyault et al., 2007; Hoshida et al., 2009; Guichard et al., 2012; Schulze et al., 2015). Over the past decade, gene signatures based on aberrant transcriptional profiles have gained widespread attention for demonstrating great promise in prognosis prediction for HCC. For example, Long et al. (2018) established a four-gene signature that could effectively recognize HCC patients at a high risk of death. Liu et al. (2018) on the other hand, identified a four differentially methylated gene pairs to predict recurrence. Several other reports also described mRNA expression signatures comprising various numbers of genes using similar approaches (Ke et al., 2018; Wang et al., 2018; Zheng et al., 2018; Chen et al., 2019; Liu et al., 2019; Qiao et al., 2019; Liu et al., 2020). However, research into molecular signatures for prognosis prediction is still in its early stage. For example, there is no consensus regarding the number and the identity of the genes taken into account by the models reported so far. Most of them displayed modest predictive capacity and were only validated retrospectively. Therefore, a considerable amount of evidence is required for this subfield to evolve and mature.

In this work, we developed a 10-gene prognostic signature using LASSO Cox regression model which outperformed previously reported HCC prognostic models and proposed a nomogram combining tumor stage and signature-defined risk group. Gene Set Enrichment Analysis (GSEA) was performed to gain a better understanding of the underlying mechanisms of our model.

## Materials and Methods

### Data Collection

For the 331 HCC patients of The Cancer Genome Atlas (TCGA) database, tumoral RNA-seq data were downloaded from the Genomic Data Commons (GDC) data portal^1^ (TCGA) and 49 of the tumors also had mRNA expression data of paired normal tissue samples. Clinical data and mutational data were downloaded using the University of Santa Cruz (UCSC) Xena^2^ and cBioPortal^3^ platforms. For the 213-patient HCC cohort of the Gene Expression Omnibus database (GEO), microarray data as well as clinical data were downloaded with an accession number of GSE14520^4^ and all of the tumors had mRNA expression data of paired normal tissue samples (Roessler et al., 2010). For the LIRI-JP cohort containing 240 HCC patients, RNA-seq data and clinical data were downloaded from the International Cancer Genome Consortium (ICGC) portal^5^. All data were downloaded from the public databases hence it was not required to obtain additional ethical approval for our study.

### Identification of Genes Differentially Expressed Between the Tumor and Normal Tissue Samples

The raw count data of the 49 paired tumor and normal samples of the TCGA cohort were normalized using the Trimmed Mean of *M*-values (TMM) method and comparative analysis was conducted using the paired *t*-test to identify differentially expressed genes (DEGs) (Robinson and Oshlack, 2010). Any gene with a false discovery rate (FDR) of <0.05 and a |log2FoldChange| higher than a cutoff as determined using the formula: mean[abs(log2FoldChange)] + 2 × sd[abs(log2FoldChange)], was regarded as a candidate DEG. Data of the GSE14520 cohort were analyzed in a similar fashion. Genes that were consistently up-regulated or down-regulated in tumor tissue in both cohorts were confirmed as DEGs.

### Development of the 10-Gene Signature

The 331-patient TCGA cohort was used as a discovery cohort to develop a gene signature for prognosis prediction. Only patients with an overall survival (OS) longer than one month were included for survival analyses. Univariate Cox regression analysis was performed to identify DEGs that are significantly associated with survival (as defined by a *P* value of <0.05). LASSO Cox regression analysis was subsequently conducted to select a panel of genes that are related with OS in HCC patients using the glmnet R package (Friedman et al., 2010). In order to select the optimal lambda parameters and corresponding coefficients in LASSO Cox regression, we performed 200 iterations of 10-fold cross-validation with binomial deviance minimization criteria on the discovery cohort. The parameters lambda via 1-SE (standard error) criteria was selected to screen for the optimal gene set. To determine the optimal gene composition, best subsets regression was adopted for gene selection (Farkas and Heberger, 2005). Finally, a 10-gene signature for predicting prognosis in TCGA discovery cohort was constructed where risk score could be calculated using the following formula:

$$\mathrm{Risk}\,\mathrm{Score}\,\left(R\,S\right)=\sum_{i}^{n}\left(E\,x\,p_{i}^{*}\,C\,o\,e\,f_{i}\right)$$

where *n* is the number of prognostic genes, *Exp*_*i*_ is the expression level of the gene, and *Coef*_*i*_ is the estimated regression coefficient of the gene.

### Validation of the 10-Gene Signature

In order to validate the predictive capacity of the signature, the TCGA discovery cohort was divided into a training dataset and a validation dataset using the createDataPartition function in the caret R package. The 240 HCC patients from the ICGC database served as a separate validation cohort. Briefly, risk score was calculated for each patient. The surv_cutpoint function in survminer R package was introduced to determine the optimal cut-off value for dissecting the population into a high-risk subset versus a low-risk subset, according to the correlation between expression levels of the signature genes and patients’ OSs in the training dataset. Kaplan–Meier (KM) survival curves combined with a log-rank test were used to test the differences in prognosis between high- and low-risk groups using the survival R package. Time-dependent receiver operating characteristic (ROC) analysis and Concordance index (C-index) were adopted to evaluate the performance of the prognostic signature for predicting 1-, 3-, and 5-year survival.

### Identification of Independent Prognostic Markers

To identify independent prognostic markers, the 10-gene signature may predict prognosis and other clinicopathological factors such as age, gender, race, body mass index (BMI), AFP, residual tumor, tumor mutational burden (TMB), tumor grade, TNM stage, and vascular tumor invasion were subjected to univariate and multivariate Cox regression analyses.

### Construction and Assessment of a Predictive Nomogram

All independent prognostic factors as revealed by multivariate analyses were combined to derive a nomogram for predicting the probability of 1-, 3-, and 5-year survival of HCC. The performance of the nomogram was evaluated using Harrell’s concordance index (C-index) and calibration curves. Decision curve analysis (DCA) was employed to compare the reliability of the nomogram with that of tumor stage or risk group alone.

### Gene Set Enrichment Analysis

Gene set enrichment analysis was performed on the high-risk and low-risk subgroups of the TCGA discovery cohort using GSEA v.3.0. Molecular Signatures Database v.7.0 was searched to identify enriched pathways associated with survival in the two risk groups, respectively. Gene sets with a *P* < 0.05 and a FDR < 25% were considered significantly enriched.

### Statistical Analyses

Statistical analyses were performed using R software v3.6.0 (R Foundation for Statistical Computing, Vienna, Austria). If not specified otherwise, tests were two-tailed, and a *P*-value of <0.05 was considered statistically significant.

## Results

### DEG Identification

**FIGURE 1:**
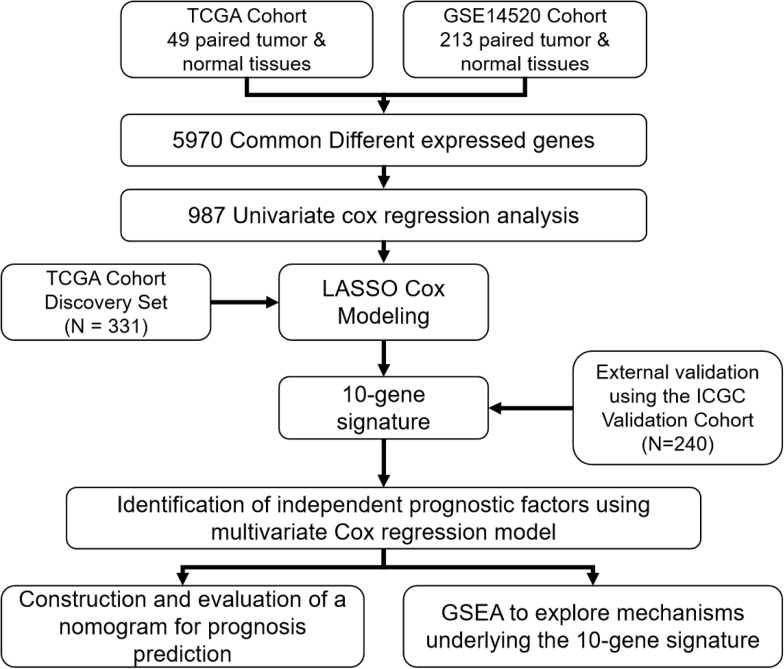
A flow chart showing the development and validation of the 10-gene signature.

A flow chart illustrating the study process is presented in Figure 1. All data were obtained from surgical tissue samples and post-operative survival data were used for all subsequent survival analyses. By analyzing the RNA-seq data of the 49 paired tumor and normal tissue samples of the TCGA cohort, the expression of 12,301 genes were found to be significantly altered (270 up-regulated and 12,031 down-regulated) in the tumor tissue. Similarly, 235 genes were up-regulated and 10,008 were down-regulated in the tumor samples according to the GSE14520 microarray dataset. By taking the intersection of the two datasets, 5,970 genes (50 up-regulated and 5,920 down-regulated) were confirmed as differentially expressed in tumor tissue (Supplementary Figure S1A).

### Development of the 10-Gene Signature

The 331 primary HCC tumors of the TCGA discovery cohort were divided into a training set (*N* = 199) and an internal validation set (*N* = 132). The baseline characteristics were summarized in Table 1. Clinicopathological features were in large balanced between the training and internal validation populations. By inputting the 5,970 DEGs identified above, a total of 987 genes were demonstrated to be significantly associated with OS for the training set using univariate Cox regression analysis. A LASSO Cox regression model was subsequently utilized to select from the 987 candidates for genes highly associated with survival as indicated by a *P* < 0.05 in univariate analyses. Twenty-six genes were identified with the lambda was 0.1 and subsequently used to construct an all subset regression model. Finally, ten genes, *YBX1, TTC26, SLC41A3, RCBTB2, PON1, MAPK7, INPP5B, CCDC134, C16orf71*, and *BMI1*, were identified to be associated with prognosis in HCC patients using the regsubsets function in leaps R package. The LASSO deviance profiles, the coefficient profile plots, and the best subset regression model were shown in Supplementary Figures S1B–D. A risk score for prognosis prediction is calculated as follows: *riskscore* = (0.921^∗^*Expr*_*YBX1*_) + (0.149^∗^*Expr*_*TTC26*_) + (0.732^∗^*Expr*_*SLC41A3*_) + (−0.631^∗^*Expr*_*RCBTB2*_) + (−0.227^∗^*Ex pr*_*PON1*_) + (0.539^∗^*Expr*_*MAPK7*_) + (1.28^∗^*Expr*_*INPP5B*_) + (0.645^∗^ *Expr*_*CCDC134*_) + (−0.478^∗^*Expr*_*C16orf71*_) + (0.861^∗^*Expr*_*BMI1*_), where *Expr* stands for the expression level of each gene. Using the training set’s survival data and the surv_cutpoint function of the survminer R package, a risk score of 0.969 was defined as the optimal cut-off value to dissect the population into a high-risk group (*N* = 100) and a low-risk group (*N* = 99), and this cut-off value was used for all subsequent stratification.

**TABLE 1:** Patient characteristics in the TCGA training and validation datasets.

**Characteristics**	**Train (*N* = 199)**	**Test (*N* = 132)**	***P*-value**
Age (IQR)	58.96(51−69)	60.33(54−68)	0.346
Gender (%)			0.526
Male	139 (69.8)	87 (65.9)	
Female	60 (30.2)	45 (34.1)	
Race (%)			0.978
Asian	89 (44.7)	58 (43.9)	
Not Asian	110 (55.3)	74 (56.1)	
AFP (%)			0.482
≥400	34 (22.1)	26 (26.8)	
<400	165 (77.9)	106 (73.2)	
BMI (%)			0.898
≥25	88 (48.6)	59 (47.2)	
<25	111 (51.4)	73 (52.8)	
Inflammation (%)			0.653
	66 (33.2)	49 (37.1)	
Mild	52 (26.1)	38 (28.8)	
None	70 (35.2)	40 (30.3)	
Severe	11 (5.5)	5 (3.8)	
Tumor_grade (%)			0.613
	3 (1.5)	2 (1.5)	
G1	26 (13.1)	25 (18.9)	
G2	98 (49.2)	56 (42.4)	
G3	65 (32.7)	45 (34.1)	
G4	7 (3.5)	4 (3.0)	
Tumor_stage (%)			0.129
Not reported	8 (4.0)	13 (9.8)	
Stage i	91 (45.7)	64 (48.5)	
Stage ii	46 (23.1)	27 (20.5)	
Stage iii	53 (26.6)	26 (19.7)	
Stage iv	1 (0.5)	2 (1.5)	
Residual_tumor (%)			0.845
	5 (2.5)	2 (1.5)	
R0	175 (87.9)	120 (90.9)	
R1	8 (4.0)	5 (3.8)	
R2	1 (0.5)	0 (0.0)	
RX	10 (5.0)	5 (3.8)	
Vascular_tumor_invasion (%)			0.404
	27 (13.6)	26 (19.7)	
Macro	11()	5 (3.8)	
Micro	52 (26.1)	29 (22.0)	
None	109 (54.8)	72 (54.5)	
TMB [mean (SD)]	5.91 (4.83)	7.70 (13.38)	0.099

### Validation of the 10-Gene Signature

**FIGURE 2:**
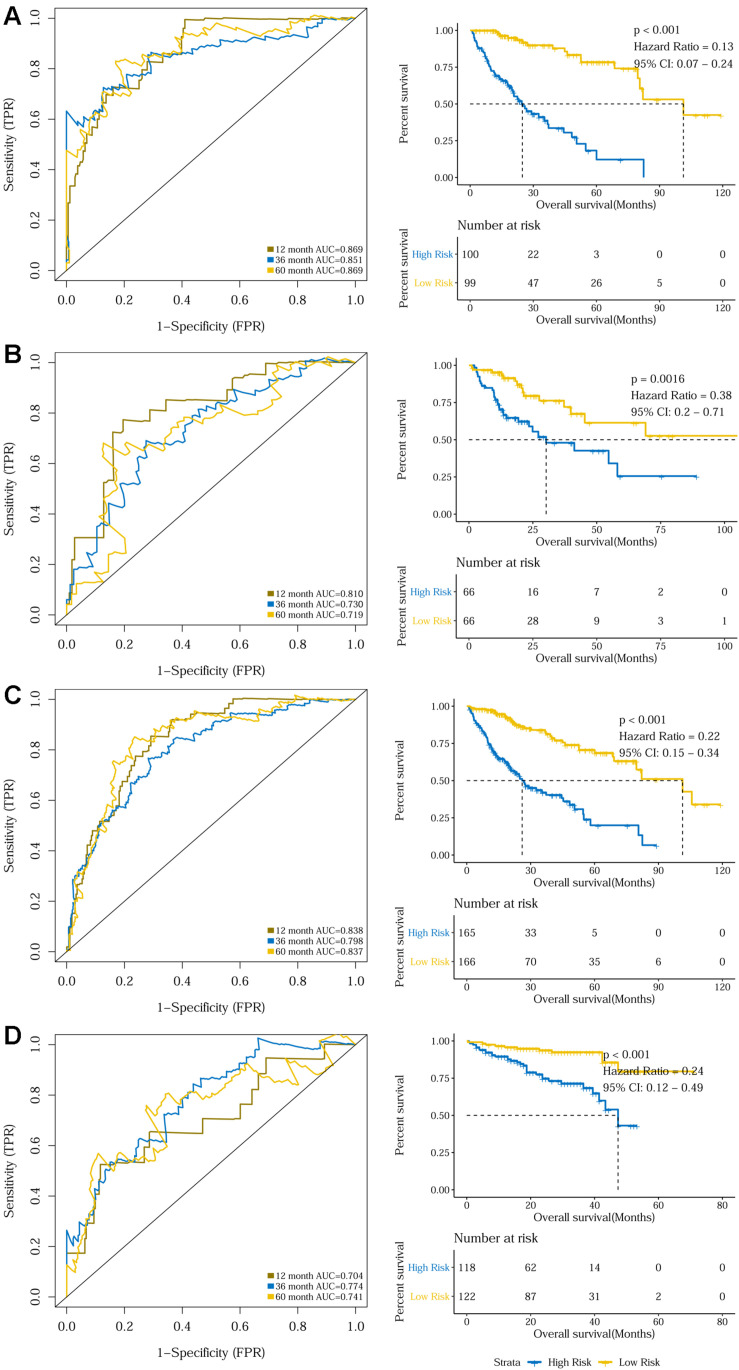
Time dependent ROC analyses and Kaplan–Meier analysis of the 10-gene signature in predicting 1-, 3-, and 5-year survival in **(A)** the training set, **(B)** the validation set, **(C)** the TCGA discovery cohort, and **(D)** the ICGC validation cohort.

The prognostic capacity of the 10-gene signature was validated in the training set, the internal validation set, as well as the entire TCGA discovery cohort, where the high-risk group included 100, 66, and 165 patients, respectively, as defined by the cut-off value of 0.969. As demonstrated by the time-dependent ROC curves, the area under the curves (AUCs) for 1-year, 3-year, and 5-year OS were 0.869, 0.851, and 0.869 for the training set, 0.810, 0.730, and 0.719 for the internal validation set, and 0.838, 0.798, and 0.837 for the entire discovery cohort, respectively. Patients in the high-risk groups in the three datasets also displayed significantly worse OSs than the low-risk groups (*P* < 0.001 for the training set, *P* = 0.0016 for the internal validation set, and *P* < 0.001 for the entire TCGA discovery cohort) (Figures 2A–C and Supplementary Figures S2A–C). Additionally, a 240-patient ICGC cohort was used as an external validation set, where 118 patients were regarded as high-risk using the same cut-off risk score. Likewise, the AUCs for the 1-year, 3-year, and 5-year OS were 0.704, 0.774, and 0.741 and the high-risk group’s prognosis was significantly worse than that of the low-risk group (*P* < 0.001) (Figure 2D and Supplementary Figure S2D). Sub-group analysis showed that the 10-gene signature remained a robust prognosis predictor across subgroups stratified according to disease stage in the discovery cohort (Supplementary Figure S3).

**FIGURE 3:**
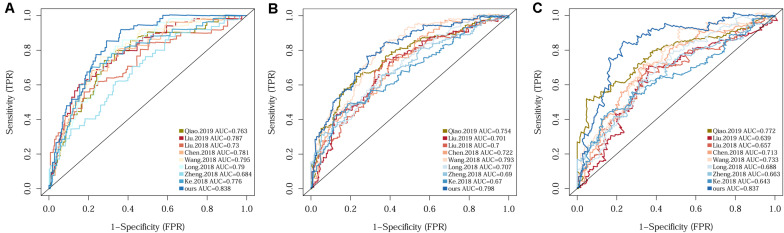
Comparison of the 10-gene signature with eight previously reported signatures using time-dependent ROC analyses for predicting 1-year **(A)**, 3-year **(B)**, and 5-year **(C)** survival.

Since several multi-gene signatures have been previously proposed for predicting HCC prognosis, their performance was evaluated in parallel to that of our 10-gene signature using time-dependent ROC curves and C-indexes. The 10-gene signature unequivocally outperformed the other eight models in terms of both C-index and the AUCs for 1-, 3-, 5-year OS prediction (Figure 3 and Supplementary Table S1).

### Identification of Independent Prognostic Markers

Univariate and multivariate Cox regression analyses were conducted on the TCGA discovery and the ICGC validation cohorts to evaluate the 10-gene signature-defined risk group as well as clinicopathological variables such as age, gender, BMI, AFP, inflammation, residual tumor, vascular tumor invasion, tumor grade, tumor stage, and TMB as independent prognosis predictors. Both risk group and tumor stage were observed to be independent prognostic factors for the discovery cohort (risk group, HR: 0.23; 95% CI: 0.14–0.36; *P* < 0.001; tumor stage, HR: 2.03; 95% CI: 1.38–3.00; *P* < 0.001) and the ICGC cohort (risk group, HR: 0.21; 95% CI: 0.08–0.56; *P* < 0.001; tumor stage, HR: 2.36; 95% CI: 1.06–5.25; *P* = 0.036). Details are provided in Table 2 and Supplementary Figure S4.

**TABLE 2:** Univariate and multivariate Cox regression to identify independent prognosis predictor in both the TCGA discovery and the ICGC validation cohorts.

**Characteristics**	**TCGA discovery cohort**					**ICGC validation cohort**				
	**Number of patients**	**Univariate mode**		**Multivariate model**		**Number of patients**	**Univariate mode**		**Multivariate model**	
		**HR (95% CI)**	***P*-value**	**HR (95% CI)**	***P*-value**		**HR (95% CI)**	***P*-value**	**HR (95% CI)**	***P*-value**
Age (≥60 vs <60)	331	1.16 (0.81–1.66)	4.20E-01							
Gender (Male vs Female)	331	0.80 (0.55–1.15)	2.30E-01			240	0.49 (0.26–0.91)	2.30E-02	0.4 (0.17–0.96)	**4.00E-02**
BMI (≥25 vs <25)	306	0.81 (0.55–1.19)	2.90E-01			240	0.87 (0.42–1.81)	7.10E-01		
AFP (≥400 vs <400)	251	1.11 (0.67–1.84)	7.00E-01							
Inflammation (Mild/Severe vs None)	216	1.28 (0.78–2.12)	3.30E-01							
Residual (R1/2 vs R0)	309	1.89 (0.95–3.74)	6.80E-02							
TMB (TMB-H vs TMB-L)	303	1.53 (0.94–2.50)	8.70E-02							
Tumor grade (G3/4 vs G1/2)	326	1.02 (0.70–1.49)	9.00E-01			240	4.55 (2.01–10.27)	**2.70E-04**	2.71 (1.17–6.29)	**2.10E-02**
Tumor stage (III/IV vs I/II)	310	2.66 (1.81–3.90)	**5.70E-07**	2.03 (1.38–3.00)	**3.60E-04**	181	2.33 (1.28–4.27)	**5.90E-03**	2.36 (1.06–5.25)	**3.60E-02**
Vascular tumor invasion (Macro/Micro vs None)	278	1.52 (0.99–2.33)	5.70E-02							
Risk (Low vs High)	331	0.22 (0.15–0.34)	**9.00E-13**	0.23 (0.14–0.36)	**2.80E-10**	240	0.24 (0.12–0.49)	**6.60E-05**	0.21 (0.08–0.56)	**1.70E-03**

*The bold values were less than 0.05.*

### Construction and Assessment of a Predictive Nomogram

**FIGURE 4:**
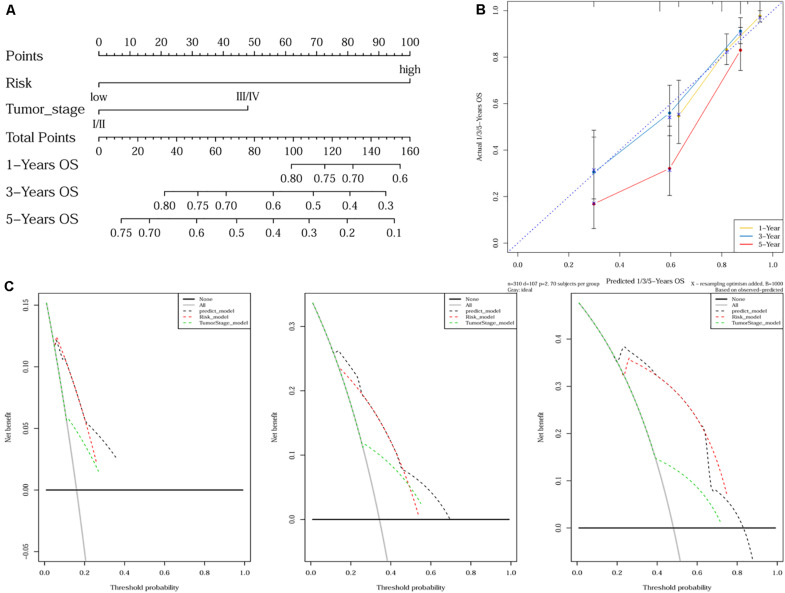
Nomogram construction and validation. **(A)** A nomogram predicting survival probability at 1-, 3- and 5-year after surgery for HCC patients; **(B)** Calibration curves for the nomogram; **(C)** DCA curves showing the comparison between the nomogram and tumor stage or risk group alone for predicting 1-, 3- and 5-year overall survival in HCC.

As tumor stage and the 10-gene signature were demonstrated to be independent prognostic factors for HCC, a nomogram incorporating tumor stage and risk group was built to predict 1-year, 3-year, and 5-year OS (Figure 4A). Calibration plots showed that the nomogram was better at predicting short-term survival (1- and 3-year) rather than long-term survival (5-year), as indicated by agreement between the predicted survival and actual survival (Figure 4B). The C-index of the nomogram (0.732, 95% CI: 0.686–0.778) was higher than that of either tumor stage (0.610, 95% CI: 0.559–0.660) or risk group (0.692, 95% CI: 0.652–0.732) alone. According to DCA curves, the nomogram also offered the highest net benefit among the three factors examined (tumor stage, risk group, and nomogram) (Figure 4C).

### Gene Set Enrichment Analyses

**FIGURE 5:**
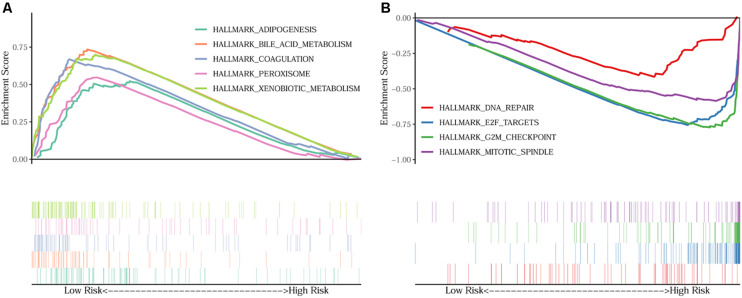
GSEA on the TCGA cohort to explore mechanisms underlying the 10-gene signature. **(A)** Five representative hallmarks in the low–risk patients; **(B)** Four representative hallmarks in the high–risk patients.

In order to unravel the molecular mechanism underlying the 10-gene signature, GSEA analysis was conducted on the TCGA discovery cohort. As shown in Table 3 and Figure 5, the G2M checkpoint (*P* = 0.002, FDR = 0.090), mitotic spindle (*P* = 0.012, FDR = 0.075), E2F targets (*P* = 0.008, FDR = 0.068), and DNA repair (*P* = 0.048, FDR = 0.125) pathways/terms were significantly enriched in the tumors of the high-risk group as defined by the 10−gene signature. In the low-risk group, the significantly enriched pathways were mainly related to various metabolic processes (including bile acid and xenobiotic metabolism), coagulation, adipogenesis, and peroxisome function.

**TABLE 3:** Significantly enriched hallmarks in the TCGA discovery cohort by GSEA.

**Name**	**Size**	**ES**	**NES**	**NOM *p*-value**	**FDR *q*-value**
HALLMARK_BILE_ACID_METABOLISM	64	0.7341	1.6979	0.00E+00	2.43E-01
HALLMARK_XENOBIOTIC_METABOLISM	122	0.6983	1.6664	1.16E-02	2.16E-01
HALLMARK_COAGULATION	67	0.6688	1.6558	5.93E-03	1.74E-01
HALLMARK_ADIPOGENESIS	92	0.5219	1.6412	3.44E-02	1.55E-01
HALLMARK_PEROXISOME	59	0.5467	1.6125	3.77E-02	1.39E-01
HALLMARK_G2M_CHECKPOINT	86	−0.7808	−1.7797	1.98E-03	9.04E-02
HALLMARK_MITOTIC_SPINDLE	83	−0.6006	−1.7329	1.17E-02	7.50E-02
HALLMARK_E2F_TARGETS	98	−0.7616	−1.7034	7.98E-03	6.82E-02
HALLMARK_DNA_REPAIR	69	−0.4284	−1.5759	4.82E-02	1.25E-01

## Discussion

Hepatocellular carcinoma (HCC) represents a major health threat worldwide, especially in East Asia. Even after curative resection, the long-term outcomes for HCC patients remain dismal. Developing a prognostic model has thus gained increasing attention. In this study, a 10-gene signature for HCC prognosis prediction was generated and validated using the mRNA expression data from three publicly accessible HCC cohorts. Of the 10 genes, only *BMI1* was positively associated with survival, while the others were all negative prognostic markers. According to risk scores calculated based on the expression profiles of the signature genes, we were able to effectively classify patients into high-risk and low-risk groups, where the low-risk subset exhibited a significantly more favorable prognosis pattern than the high-risk group. This 10-gene signature also proved to be an independent prognosis factor for HCC survival. A nomogram combining both tumor stage and risk group was proposed, which proved to be a better predictor than tumor stage or risk group alone. Additionally, it was noteworthy that patients in the low-risk group were more closely associated with alterations in metabolic pathways, while the high-risk group were more enriched in cell proliferation-related pathways such as G2M checkpoint.

*YBX1* is a widely known oncogene implicated in multiple malignancies (Xu et al., 2017). Accumulating evidence has shown that aberrant *YBX1* expression is closely associated with tumor progression, drug resistance, metastasis and poor prognosis in cancers (Imada et al., 2013; Davies et al., 2014; Kosnopfel et al., 2014; Wu et al., 2015). In HCC, the expression of *YBX1* is activated by lncRNAs, which in turns regulates the PIK3CA pathway (Zhao et al., 2017). The *TTC26* gene encodes an intraflagellar transport protein, which transports motility-related proteins into flagella (Ishikawa et al., 2014). Wang et al. (2018) built a six-gene signature, of which *TTC26* was also a positive prognostic gene. However, the exact role of *TTC26* in HCC remains unclear. *SLC41A3* (Solute Carrier Family 41 Member 3) encodes a protein with cation transmembrane transporter activity that may contribute to Mg^2+^ transportation (de Baaij et al., 2016). THPA (The Human Protein Altas) database showed that HCC patients had the lowest expression level of *SLC41A3* across different cancer types, and high-expression group had significantly worse survival. Nevertheless, the role of *SLC41A3* in HCC remains largely undefined. *RCBTB2* (regulator of chromosome condensation and BTB domain containing protein 2) also known as *CHC1L*, has been proposed as a tumor suppressor gene in prostate cancer (Latil et al., 2002). An earlier study evaluated the relationship between *RCBTB2* expression and carcinogenesis of multiple myeloma, and a low expression of *RCBTB2* is linked to pathogenesis and progression of multiple myeloma (Legartova et al., 2010). However, *RCBTB2* gene has not never been reported in association with HCC to date. *PON1* is a member of the paraoxonase family, and it is an antioxidant defensive factor that is relevant for the pathogenesis of several inflammatory diseases (Mackness and Mackness, 2015; Borovkova et al., 2017). A growing body of evidence has suggested that *PON1* could serve as an important clinical indicator of cancer progression for a number of cancers such as lung cancer and breast cancer (Bobin-Dubigeon et al., 2015; Aldonza et al., 2017). Data in THPA database showed that the expression of *PON1* in HCC is significantly higher than that in other tumors, and its down-regulated expression has been implicated to be a poor indicator for survival in patients with HCC (Yu et al., 2018). *MAPK7* (mitogen-activated protein kinase 7) encodes extracellular-regulated protein kinase 5 (ERK5). Zen et al. (2009) suggested that *MAPK7* is a probable target of 17p11 amplification and that the ERK5 protein product of *MAPK7* gene promotes the growth of HCC cells by regulating mitotic entry. *INPP5B* (inositol polyphosphate-5-phosphatase B) encodes an inositol polyphosphate-5-phosphatase and regulates calcium signaling by inactivating inositol phosphates. *OCRL1* is a homologue of *INPP5B*, shared the same domain structure and substrate specificity (Lowe, 2005). As yet it is not clear whether *INPP5B* is related to HCC. MAPK cascades are critical signaling pathways involved in regulation of cellular processes such as growth, proliferation, differentiation, migration, invasion, and apoptosis (Dhillon et al., 2007). Previous studies showed that *CCDC134* (coiled coil domain containing 134) acts as an inhibitor of Erk1/2 and JNK/SAPK pathways and its silencing promotes cell migration and invasion in cancers such as gastric cancer (Liang et al., 2005; Zhong et al., 2013). However, no such evidence has been found in HCC. *BMI1* is recognized as one of the most commonly activated oncogenes in various tumor types, including prostate, colorectal and lung cancers (Kim et al., 2004; Vrzalikova et al., 2008; Ganaie et al., 2018). The over-expression of *BMI1* correlates with therapy failure in breast, prostate, lung cancer and HCC patients (Glinsky, 2007; Vrzalikova et al., 2008; Wang et al., 2008). Recent studies demonstrated that increased expression of *BMI1* resulted in therapy failure and indicated poor prognosis of HCC (Wang et al., 2008; Ruan et al., 2013).

While constructed on cohorts mainly comprising Caucasian HCC patients, our 10-gene signature was also shown to be a reliable predictor of prognosis among Asian patients in the ICGC cohort and the cut-off risk score for differentiating patient’s risk of death, as trained using the TCGA cohort, could be directly applied to other populations. Compared to several existing multi-gene models, our signature also demonstrated better performance in distinguishing patients at high risk. This might be partly attributed to the fact that in our study, both model construction and validation were carried out using RNA-seq data (although microarray data of the GEO database served as a supplementary source to confirm DEGs identification in the beginning), while most of the other signatures were developed and validated using data generated on different platforms, RNA-seq for development and microarray for validation or *vice versa*. Another explanation could be that our DEGs selection was strictly based on paired tumor and normal tissue samples obtained from the same patients, which was obviously not the case in most other studies. However, our study do have some limitations. First, this was a retrospective analysis based on public datasets, therefore should be viewed as hypothesis generating rather than conclusive. Second, the validity of our signature could potentially be challenged by the heterogeneity of HCC due to sampling bias. Therefore, the 10-gene signature may warrant further prospective validation and the correlation between expression levels of these genes at protein level and patients’ prognosis is also worth exploring.

## Conclusion

Collectively, we established a robust 10-gene signature and a nomogram to predict OS of HCC patients, which may help recognize high-risk patients potentially benefiting from more aggressive treatment.

## Data Availability Statement

Publicly available datasets were analyzed in this study. The data are accessible at the following repositories: https://portal.gdc.cancer.gov/repository, https://www.ncbi.nlm.nih.gov/geo/query/acc.cgi?acc=GSE14520, and https://dcc.icgc.org/projects/ LIRI-JP.

## Author Contributions

YT: conceptualization. TZ and ZC: formal analysis and validation. MH, YB, and YN: investigation. TZ: writing – original draft. NM, WX, CG, and YN: writing – review and editing. All authors have read and agreed to the published version of the manuscript, contributed to the article and approved the submitted version.

## Conflict of Interest

WX, CG, MH, and YB were employed by company 3D Medicines Inc., China. The remaining authors declare that the research was conducted in the absence of any commercial or financial relationships that could be construed as a potential conflict of interest.

## Footnotes

1 https://portal.gdc.cancer.gov/

2 https://xena.ucsc.edu/

3 http://cbioportal.org/

4 https://www.ncbi.nlm.nih.gov/geo/query/acc.cgi?acc=gse14520

5 https://dcc.icgc.org/projects/LIRI-JP
