# Supplementary material for: A Novel Ten-Gene Signature Predicting Prognosis in Hepatocellular Carcinoma
Source: Front Cell Dev Biol. 2020 Jul 14;8:629. doi: 10.3389/fcell.2020.00629 (PMC7372135; doi:10.3389/fcell.2020.00629)
Supplement: Supplementary file 1 [file Data_Sheet_1.docx]

Supplementary Material

# Supplementary Figures and Tables

## Supplementary Figures


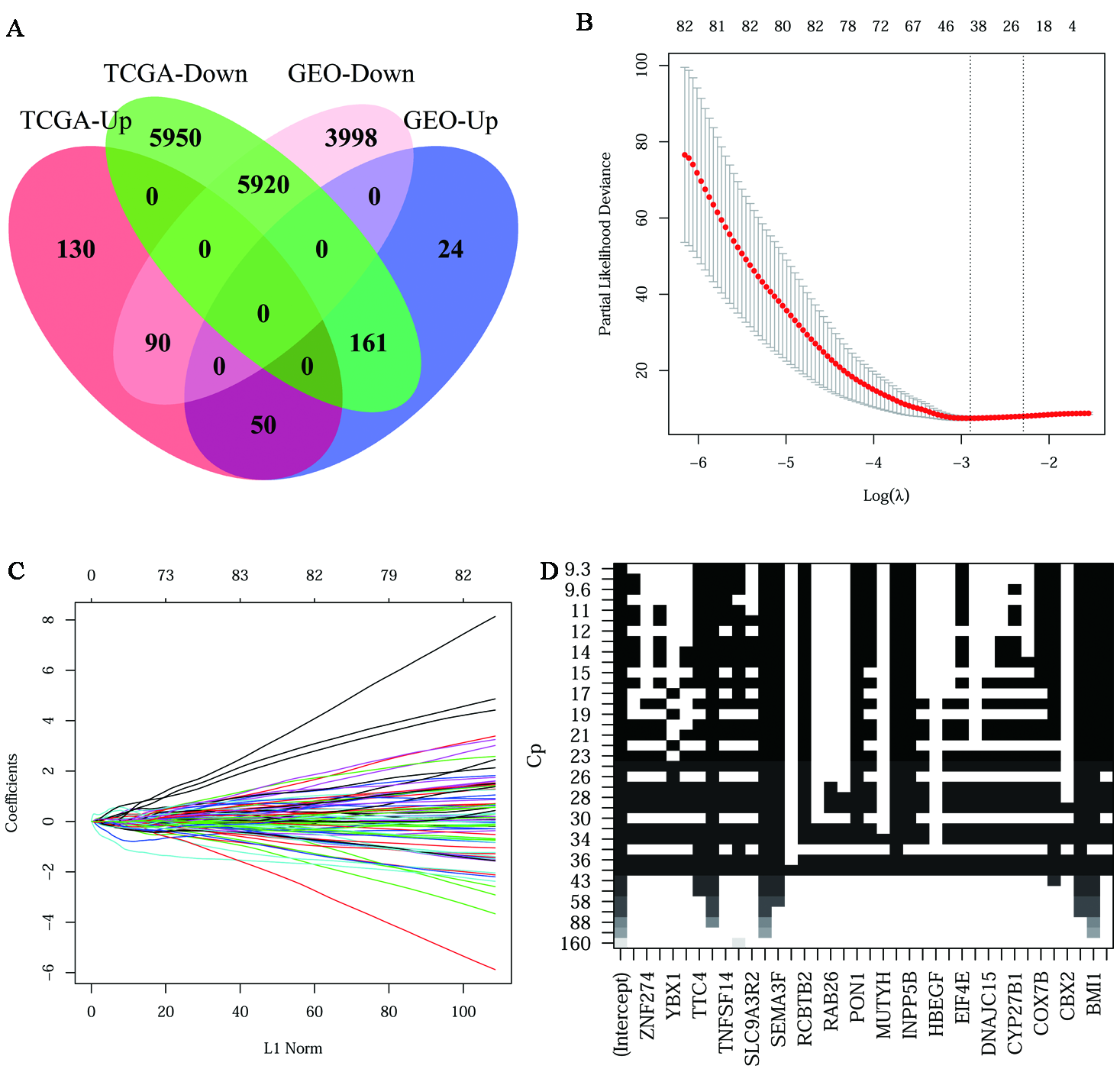


**Supplementary Figure 1**. A) Identification of commonly up-regulated and down-regulated genes in both the TCGA and GEO cohorts; B) LASSO deviance profiles of the cancer-associated mRNAs in HCC; C) LASSO coefficient profiles of the cancer-associated mRNAs in HCC; D) The optimal subsets selection using best subset regression model.


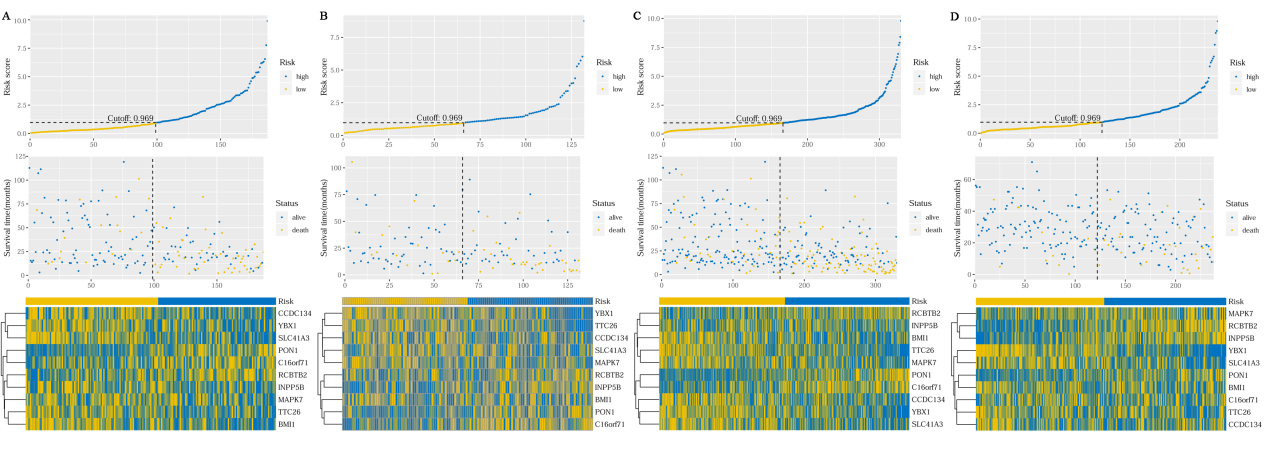


**Supplementary Figure 2**. The distribution of risk scores, risk groups and survival statuses in A) the training set, B) the validation set, C) the TCGA discovery cohort and D) the ICGC validation cohort.


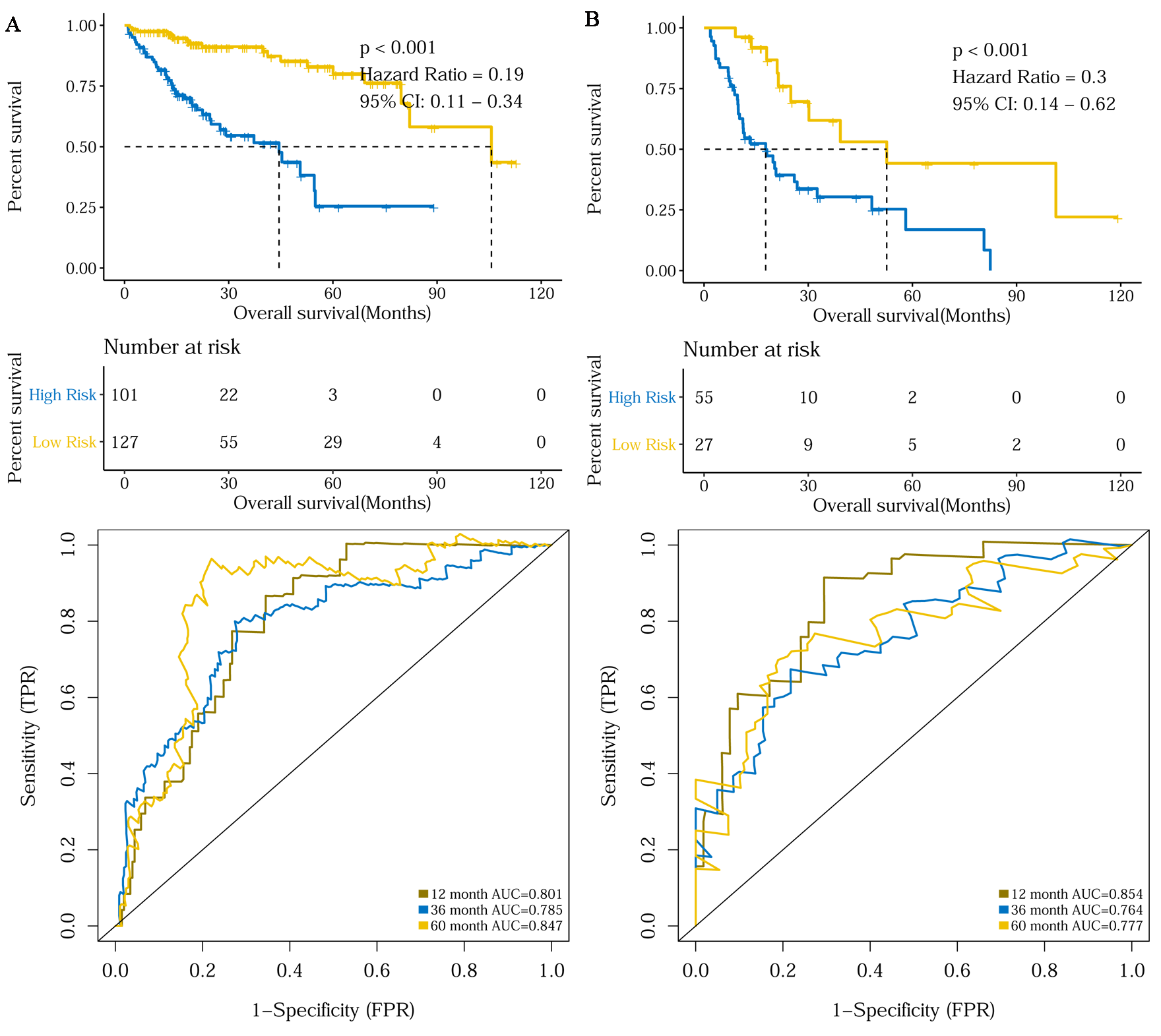


**Supplementary Figure 3**. Kaplan–Meier analysis (low risk vs. high risk) and time-dependent ROC analyses (1-, 3-, and 5-year) of subgroups stratified by tumor stage. A) tumor stage I/II; B) tumor stage III/IV.


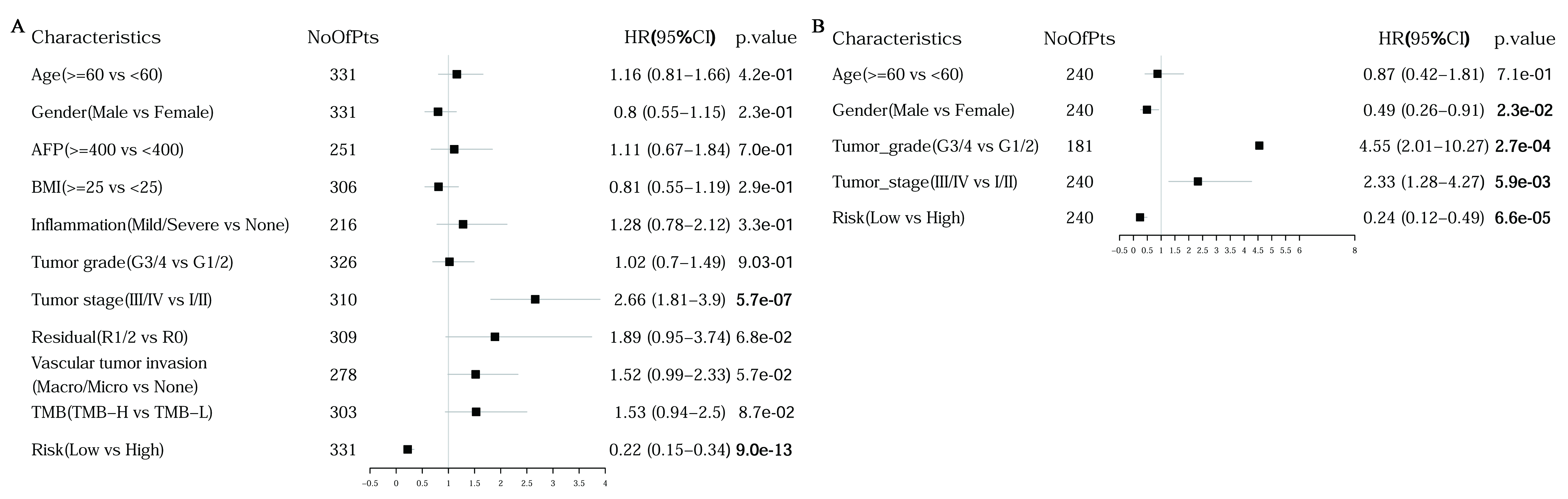


**Supplementary Figure 4**. Forrest plots of univariate Cox regression analysis to identify independent prognosis predictors in A) the TCGA discovery cohort and B) the ICGC validation cohort.

## Supplementary Tables

**Supplementary Table 1** Comparison of the 10-genesignature with eight other previously reported signatures.

| **Models** | **C-index (95% CI)** | **1-year AUC (95% CI)** | **P.value** | **3-year AUC (95% CI)** | **P.value** | **5-year AUC (95% CI)** | **P.value** |
| --- | --- | --- | --- | --- | --- | --- | --- |
| Ours | 0.76(0.72-0.81) | 0.84(0.79-0.89) |  | 0.81(0.74-0.87) |  | 0.85(0.78-0.93) |  |
| Qiao.2019 | 0.73(0.68-0.78) | 0.77(0.70-0.84) |  | 0.75(0.68-0.82) |  | 0.76(0.68-0.84) |  |
| Liu.2019 | 0.71(0.66-0.76) | 0.79(0.72-0.86) |  | 0.74(0.66-0.81) |  | 0.69(0.59-0.78) |  |
| Liu.2018 | 0.69(0.63-0.74) | 0.73(0.65-0.82) |  | 0.72(0.64-0.79) |  | 0.70(0.61-0.80) |  |
| Chen.2018 | 0.73(0.68-0.77) | 0.79(0.72-0.85) |  | 0.73(0.65-0.80) |  | 0.73(0.63-0.82) |  |
| Wang.2018 | 0.73(0.68-0.77) | 0.79(0.73-0.86) |  | 0.80(0.73-0.86) |  | 0.78(0.70-0.87) |  |
| Long.2018 | 0.73(0.68-0.77) | 0.79(0.73-0.85) |  | 0.72(0.64-0.79) |  | 0.70(0.59-0.80) |  |
| Zheng.2018 | 0.67(0.62-0.73) | 0.68(0.60-0.76) |  | 0.70(0.62-0.78) |  | 0.67(0.57-0.77) |  |
| Ke.2018 | 0.70(0.65-0.75) | 0.78(0.71-0.85) |  | 0.68(0.60-0.76) |  | 0.67(0.57-0.76) |  |
| Ours vs Qiao.2019 |  |  | 0.0888 |  | 0.1868 |  | 0.0527 |
| Ours vs Liu.2019 |  |  | 0.1052 |  | 0.0574 |  | 0.0002 |
| Ours vs Liu.2018 |  |  | 0.0054 |  | 0.0192 |  | 0.0022 |
| Ours vs Chen.2018 |  |  | 0.0857 |  | 0.0314 |  | 0.0043 |
| Ours vs Wang.2018 |  |  | 0.1244 |  | 0.7796 |  | 0.1552 |
| Ours vs Long.2018 |  |  | 0.0917 |  | 0.0169 |  | 0.0012 |
| Ours vs Zheng.2018 |  |  | 0.0001 |  | 0.0099 |  | 0.0003 |
| Ours vs Ke.2018 |  |  | 0.0213 |  | 0.0011 |  | 0.0002 |
